# Psychometric property and measurement invariance of internet addiction test: the effect of socio-demographic and internet use variables

**DOI:** 10.1186/s12889-022-13915-1

**Published:** 2022-08-13

**Authors:** Xi Lu, Kee Jiar Yeo, Fang Guo, Zhenqing Zhao, Ou Wu

**Affiliations:** 1 Hangzhou Vocational &Technical College, Zhejiang, 310018 Hangzhou China; 2 Department of Education, Universiti Teknologi Malaysia, 81310 UTM, Johor Bahru, Malaysia; 3 Shulan International Medical College, Zhejiang Shuren University, Zhejiang, People’s Republic of China

**Keywords:** Internet addiction test (IAT), Pathological internet use (PIU), College students

## Abstract

**Background:**

According to the validation literature on items of Young’s Internet Addiction Test (IAT), this study rephrased disputable items to improve the psychometric properties of this Chinese version of IAT and identify the presence of differential item function (DIF) among demographic and Internet use factors; detect the effect of demographic and Internet use factors on IAT after adjusting for DIF.

**Methods:**

An online questionnaire was distributed to college students in Zhe Jiang province in two stage. The 1st phase study collected 384 valid responses to examine the quality of IAT items by using Rasch Model analysis and exploring factor analysis (EFA). The online questionnaire was modified according to the 1st phase study and distributed online for the 2nd phase study which collected a total of 1131 valid responses. The 2nd phase study applied confirmatory factor analysis (CFA) and a multiple indicator multiple causes (MIMIC) model to verify the construct of IAT, potential effect of covariates on IAT latent factors, as well as the effect of differential item functioning (DIF).

**Results:**

Rasch model analysis in the 1st phase study indicated a 5-point rating scale was performed better, no sever misfit was found on item. The overall property of Chinese version IAT with the 5-point scale was good to excellent person and item separation (2.66 and 6.86). A three-factor model was identified by EFA. In the 2nd phase study, IAT 13 were detected with DIF for gender in MIMIC model. After correcting DIF effect, the significant demographic and Internet use factors on IAT were time spent online per day, year 3, year 2, general users.

**Conclusion:**

Item improvement was efficient that the problematic items found in literature was performed good in this study. The overall psychometric property of this Chinese version IAT was good with limited DIF effect in one item. Item improvement on IAT13 was encouraged in the future study to avoid gender bias and benefit for epidemiology on PIU.

The development of smartphone and 5G technology make it easy to access Internet and change people’s life in China, such as online payment, consumer behaviour. Internet become an important part of people’s life. The 47th report from China Internet Network Information Center [1] indicated that up to December 2020, there were 989 million Internet users in China who spent 26.2 hours weekly online, 17.1% of users were under the age of 20. Most of them (99.7%) used smartphone to get Internet. Game Apps were the top App category among the top four categories in the market, accounting for 25.7% of all Apps. The adverse effect of Internet overuse was evident, such as poor academic performance, psychological and physical health problems [2–6]. In China, Internet overuse becomes a public health concern, especially on college students [7, 8]. There were a few different terms to describe the phenomenon of maladaptive Internet use including “Internet addiction, Internet addicts, Pathological Internet use, Internet Addiction Disorder, Problematic Internet use, maladaptive patterns of Internet use, computer-medicated communication addicts, computer junkies, etc.” [9–13]. In this study, the term “Pathological Internet use” (PIU) was taken to describe the behaviour of inability to control Internet use that would in turn lead to physical, psychological and social problems, affect individual’s social function and daily life [10, 11, 14].

The prevalence of PIU on college students was varied among different countries ranged from 3.2 to 43% [15–21]. Despite the sample difference, the inconsistent measurement instrument and cut-off point might contribute to the great discrepancy on prevalence rate of PIU [15]. A review study on the existing measurement tool of Internet addiction found that there were 45 tools to measure PIU, but most of them were not well-validated [22]. A valid assessment tool is important for clinical and research setting. Exploring the psychometric properties of existing tool in diverse culture and age group was deemed more efficient, rather than developing a new scale [14, 22, 23].

Young’s Internet Addiction Test (IAT) was found to be the most validated and frequently used instrument among studies in different countries [15, 22]. It was well validated in 17 languages, such as English, Chinese, Italian, Greek, Korean, Thai, French, Turkish, Malay [14, 22, 24, 25]. It was also one of the most frequently used instruent to examin the prevalence of PIU in China [15]. The result of construct validity on factor analysis was varied which found 1 to 6 factor models [20, 22, 26–33]. Previous validation study on bilingual version of IAT found some problematic items, such as IAT7, IAT11 [31], IAT 3, IAT9 [34]. The expression or translation of some items should be upgrade or reformulated [22]. This study was aimed to rephrase the Chinese version of IAT and examine the item-level psychometric properties in a sample of college students in order to upgrade the construct quality of IAT under Chinese background. The effect of socio-demographic and Internet use factors on IAT was also identified after controlling the differential item function (DIF).

## Methods

### Participants and procedure

This study was carried in two phases, which used different samples of three-year college students in Zhejiang, China. In the first phase, a total of 384 students from Hangzhou Vocational &Technical College were answered the questionnaire in order to examine the validity of IAT items. There were 208 males and 140 females at the age of 18.34 (SD = 0.76), 184 students were the only child in the family (Table 1).

**Table 1:** Characteristics of 1st phase study sample

	n or Mean	SD or %
Gender		
Male	208	59.77
Female	140	40.23
Only child		
Yes	184	52.87
No	164	47.12
Age (year)	18.34	0.76

In the second phase, data were collected from four colleges (Zhejiang Institute of Mechanical & Electrical Engineering, Wenzhou Vocational College of Science & Technology, Hangzhou Vocational &Technical College, Zhejiang Yuying College of Vocational and Technology). As shown in Table 2, a total of 1131 students participated in the 2nd phase study, 598 were male and 533 were female. There were 408 from 1st year, 488 from 2nd year, 235 from 3rd year. The number of respondents from four major filed was roughly equivalent (344 from art, humanity and social science, 238 from science, 229 from engineering, 320 from others). Students were divided into five Internet use groups according to their respond on favorite online activity, who rate the MMORPG as their favorite activity were deemed as MMORPG users (*n* = 229), rate cellphone game as the favorite activity were cellphone game users (*n* = 158), choose SNS as favorite activity were SNS users (*n* = 422), who generally try various online activities and do not have favorite activity were deemed as general users (*n* = 179). The other users (*n* = 143) were those who have favorite Internet activity, but were neither SNS nor game, such as online searching, shopping, video, gambling etc.

**Table 2:** Characteristics of 2nd phase study sample

Categorical /Ordinal measures	N	%	Continuous measures	N	Mean	S.D.
Gender			age	1131	20.05	2.43
Male	598	52.87	time spent online	1131	5.66	2.82
female	533	47.13	experience	1131	11.31	2.72
Programme			time spent on favorite app	1131	3.75	2.05
1st year	408	36.07				
2nd year	488	43.15				
3rd year	235	20.78				
Major						
Art, humanity and social science	344	30.42				
science	238	21.04				
engineering	229	20.25				
others	320	28.29				
Internet use group						
General users	179	15.83				
MMORPG uses	229	20.25				
Cellphone game users	158	13.97				
SNS users	422	37.31				
Other users	143	12.64				

### Measure

The questionnaire used in this study comprised two parts, first is the basic information of college students including gender, major field, time spent online, and years of Internet use experience; second part is the Internet Addiction Test (IAT) which is a 20-item of self-report instrument used to measure the individual’s Internet use from the perspective of psychological symptoms and behaviors, such as psychological dependence, compulsive use, and withdrawal, problems of school, sleep, family, and time management. It was developed based on Young’s YDQ [13, 14]. The original English version of IAT was translated into Chinese using translation and back translation procedures. Phrases were modified to adapt to current internet use situation and sample background, such as in item 6, “grades/coursework/study” replaced the word “work”; “email” in item 7 was changed to “online instant message (e.g. qq, wechat). The first version was scored on a 5-poin Liker scale, 1 for rarely, 2 for occasionally, 3 for frequently, 4 for often, 5 for always. It was modified In Young and Nabuco de Abreu’s latest book “Internet Addiction: A Handbook and Guide to Evaluation and Treatment”, the items are rated on a 6-point scale regarding to participants’ experience of their Internet use: 0 for not applicable, 1 for rarely, 2 for occasionally, 3 for frequently, 4 for often, 5 for always. The cut-off point for severe Internet addiction was 70–100 and 80–100 respectively. This study chose the latest scoring method (6-point rating scale) for IAT items.

### Statistical analyses

In the 1st phase study, Rasch model analysis was first applied to examine unidimensionality assumption, rating scale property, item fit and reliability by Winsteps version 3.75.0. Principal components analyses of residuals (PCA) was used to test unidimensionality, which the raw variance explained by measures should be more than 40% and the unexplained variance explained by 1st contrast should be less than 2 eigenvalue [35]. Category structure was tested to examine the monotonic ordering of 6-category rating scale. Mean square standardized residuals (MNSQ) of INFIT and OUTFIT were indices of item fit, the value between 0.5 to 1.5 is deemed productive [36]. Separation coefficient is the signal-to-noise ratio, the ratio of “true” variance to error variance. The person reliability is equivalent to KR-20, Cronbach Alpha Coefficient. And the item reliability is equivalent to construct validity [37]. Second, exploratory factor analysis (EFA) was conducted to identify the construct of IAT by Mplus version 6 using WLSMV estimator [38].

In the 2nd phase study, the construct of IAT was verified by confirmatory factor analysis (CFA). The differential item functioning (DIF) and the effect of covariates on IAT latent factors were examined by a multiple indicator multiple causes (MIMIC) model. The covariates in the MIMIC model were Internet use variables and socio-demographic variables (Table 1). The Internet use variables included years of Internet use experience (M = 11.31, SD = 2.72), time spent online per day (M = 5.66 h, SD =2.82), favorite Internet activate (general users as the reference group). The socio-demographic variables were age (M = 20.05 years, SD = 2.43), programme (3rd year as reference group), gender (male as reference group), and major (art, humanity and social science as reference group).

Numbers of model fit indices were found in Mplus. This study used RMSEA, CFI, TLI, SRMR for model fit evaluation [39]. Root Mean Square Error of Approximation (RMSEA) was suggested that the value less than 0.05 was good fit, blow 0.08 and above 0.05 as acceptable fit. The Standardized Root Mean Square Residual (SRMR) was suggested to be in the range of 0.05 and 0.10 as acceptable, between 0 and 0.05 as good fit [39]. The Comparative Fit Index (CFI) value above 0.95 was considered as good fit, and greater than 0.90 as acceptable fit [40]. The Tucker-Lewis Index (TLI) also known as the Nonnormed Fit Index (NNFI), which the value above .90 were considered as acceptable fit, and above .95 as good fit [40].

## Result

### 1st phase study

The 1st phase study sample (*n* = 348) was used to test the item quality and validity of IAT. Correction may necessary if it helps to meet the required psychometric property of instrument. Rasch analysis was first used to evaluate the category rating scale and item property. The construct validity of IAT was identified by exploratory factor analysis (EFA).

The result of Rasch principal component analysis (PCA) in Table 3 showed that the raw variance explained by measure was 43% and unexplained variance in 1st contrast was 5.5% with 1.9 eigenvalue indicating that the IAT showed a good fit as a unidimensional scale.

**Table 3:** IAT Standardized residual variance (in Eigenvalue units) (*n* = 348)

Total raw variance in observations	Empirical		Modeled
	35.1	100.0%	100.0%
Raw variance explained by measures	15.1	43.0%	43.4%
Raw variance explained by persons	5.0	14.2%	14.3%
Raw Variance explained by items	10.1	28.8%	29.1%
Raw unexplained variance (total)	20.0	57.0%	56.6%
Unexplned variance in 1st contrast	1.9	5.5%	9.7%
Unexplned variance in 2nd contrast	1.5	4.3%	7.5%

Category structure was evaluated, which found disordered threshold of structure calibration between 1 (rarely) and 2 (occasionally) response (Table 4). Therefore, an original 6-category rating scale was converted to a 5-category rating scale by collapsing 1 (rarely) and 2 (occasionally) response. As shown in Table 4, the value of structure calibration increases with the category value, and the new category system performed better than the 6-category system. The overall property of IAT with 5-category rating scale showed a good to excellent person and item separation (2.66 and 6.86) (Table 4).

**Table 4:** Summary of category structure on IAT 6- and 5- category rating scale (*n* = 348)

PERSON SEPARATION (RELIABILTIY)	ITEM SEPARATION (RELIABILTIY)	CATEGORY		OBSERVED		OBSVD AVRGE	SAMPLE EXPECT	INFIT MNSQ	OUTFIT MNSQ	STRUCTURE CALIBRATN	CATEGORY MEASURE	
		LABEL	SCORE	COUNT	%							
2.44 (.86)	7.44 (.98)	0	0	2871	41	−2.10	−2.09	.98	.99	NONE	(− 2.74)	0
		1	1	1164	17	−1.50	−1.53	.98	.90	−.95	1.54	1
		2	2	1954	28	−1.05	− 1.04	1.01	1.00	−1.80	−.58	2
		3	3	688	10	−.51	−.56	.94	.95	.24	.41	3
		4	4	238	3	−.13	−.10	1.07	1.06	.73	1.49	4
		5	5	45	1	−.08	.32	1.40	1.37	1.78	(3.10)	5
2.66 (.88)	6.86 (.98)	0	0	2871	41	−3.06	−3.02	.94	.97	NONE	(−3.67)	0
		1	1	3118	45	−1.77	−1.80	.98	.93	−2.53	−1.31	1
		2	2	688	10	−.80	−.87	.95	.95	.19	.25	2
		3	3	238	3	−.28	−.17	1.11	1.22	.55	1.40	3
		4	4	45	1	−.15	.37	1.43	1.77	1.77	(3.06)	5

Table 5 is the item fit statistics in misfit order, which showed that all the point-measure correlation (CORR.) are positive and high, ranged from 0.41 to 0.63, all are close to the expected correlation (EXP.). It implied that all the items are aligned with the abilities of person. The average item infit and outfit MNSQ is close to 1, ranged from 0.71 to 1.48.

**Table 5:** Item fit statistics of IAT in misfit order (n = 348)

ENTRY NUMBER	TOTAL		MODEL		INFIT		OUTFIT		PT-MEASURE		EXACT MATCH		
	SCORE	COUNT	MEASURE	S.E.	MNSQ	ZSTD	MNSQ	ZSTD	CORR.	EXP.	OBS%	EXP%	ITEM
4	652	348	−.32	.08	1.31	3.3	1.48	4.9	A .41	.58	50.0	58.0	iat4
1	804	348	−1.22	.07	1.23	2.7	1.32	3.7	B .57	.64	50.3	52.0	iat1
12	704	348	−.66	.08	1.26	2.8	1.22	2.4	C .63	.60	49.4	55.6	iat12
9	585	348	.19	.09	1.16	1.7	1.20	2.1	D .47	.54	60.6	61.8	iat9
7	512	348	.88	.10	1.19	2.0	1.10	.9	E .46	.49	65.3	67.3	iat7
11	608	348	.00	.09	1.15	1.7	1.12	1.3	F .58	.55	57.6	60.5	iat11
19	569	348	.32	.09	1.12	1.3	1.05	.5	G .55	.53	66.2	63.0	iat19
3	500	348	1.01	.11	1.10	1.1	1.03	.3	H .50	.47	69.4	68.5	iat3
18	496	348	1.06	.11	1.08	.9	.96	−.3	I .50	.47	68.8	68.8	iat18
20	495	348	1.07	.11	1.06	.7	.91	−.8	J .52	.47	75.0	68.9	iat20
5	737	348	−.85	.08	.98	−.2	1.03	.4	j .57	.62	58.2	53.7	at5
17	611	348	−.02	.09	1.03	.4	.99	−.1	i .57	.56	64.4	59.9	iat17
14	611	348	−.02	.09	1.00	.1	.99	.0	h .55	.56	62.1	59.9	iat14
16	678	348	−.49	.08	.96	−.5	.95	−.6	g .62	.59	60.6	56.7	iat16
10	617	348	−.07	.09	.92	−1.0	.89	−1.3	f .61	.56	56.5	59.5	iat10
2	646	348	−.28	.08	.78	−2.7	.85	−1.8	e .61	.57	65.6	58.0	iat2
6	678	348	−.49	.08	.80	−2.5	.84	−1.9	d .65	.59	62.4	56.7	iat6
8	601	348	.06	.09	.75	−3.1	.79	−2.5	c .59	.55	65.0	60.6	iat8
15	665	348	−.41	.08	.70	−3.8	.77	−3.0	b .59	.58	65.9	57.2	iat15
13	579	348	.24	.09	.71	−3.6	.73	−3.2	a .59	.54	68.8	62.1	iat13
MEAN	617.4	348.0	.00	.09	1.01	.1	1.01	.1			62.1	60.4	
S.D.	80.1	.0	.62	.01	.18	2.1	.19	2.1			6.7	4.8	

As previous research have found one- to six- factor solutions for IAT, this research identified the one- to six- factor models respectively in Mplus. As shown in Table 6, a 3-factor model was found to be fit better and acceptable (x^2^ /df < 2, RMSEA = 0.031, SRMR = .037, CFI = .991, TLI = .988), all factor loadings were above 0.30 and significant, factors were correlated moderately to high (r = 0.541–0.774). The cut-off point of loadings was low in order to compute item loadings for further inspection in CFA analysis. A cross-loading was found on iat18. As the loading on factor 2 is much higher than loading on factor 3, iat18 was grouped in factor 2. Factor 1 had five items (iat1, iat2, iat5, iat6, iat8) that related to time management problem and negative influence on study/job of Internet use. Factor 2 is consists of 11 items (iat10, iat11, iat12, iat13, iat14, iat15, iat16, iat17, iat18, iat19, iat20) that measure the excessive use and emotional conflict of Internet use. Factor 3 contains four items (iat3, iat4, iat7, iat9) relating to neglect social life of Internet use.

**Table 6:** Factor loadings, factor Correlations of EFA for IAT (*n* = 348)

Items		Factor loading			
			1	2	3
IAT1: find that you stay online longer than you intended			0.765		
IAT2: neglect household chores to spend more time online			0.738		
IAT3: prefer the excitement of the Internet to intimacy/relationships with your partner/friends					0.441
IAT4: form relationships with fellow online users?					0.303
IAT5: others in your life complain to you about the amount of time you spend online			0.626		
IAT6: your grades/coursework/study suffer because of the amount of time you spend online			0.801		
IAT7: check your instant message (e.g. qq, wechat) before something else that you need to do?					0.427
IAT8: your study performance or productivity suffer because of the Internet use			0.392		
IAT9: become defensive or sensitive when anyone asks you what you do online?					0.424
IAT10: block out disturbing thoughts about your life with soothing thoughts of the Internet				0.469	
IAT11: find yourself anticipating when you will go online again				0.927	
IAT12: fear that life without the Internet would be boring, empty, and joyless				0.724	
IAT13: snap, yell, or act annoyed if someone bothers you while you are online				0.816	
IAT14: lose sleep due to late-night Internet use				0.339	
IAT15: feel preoccupied with the Internet when offline, or fantasize about being online				0.763	
IAT16: find yourself saying “just a few more minutes” when online				0.660	
IAT17: try to cut down the amount of time you spend online and fail				0.329	
IAT18: try to hide how long you’ve been online				0.429	0.367
IAT19: choose to spend more time online over going out with others				0.529	
IAT20: feel depressed, moody or nervous when you are offline, which goes away once you are back online				0.502	
factor correlations					
1	1.000				
2	0.774	1.000			
3	0.541	0.630	1.000		

*Notes: All factor loadings are significant at p < 0.01*

### 2nd phase study

The 2nd phase study was conducted on a sample of 1131 college students, which is aimed to verify the structural validity of IAT found in the 1st phase study, test the DIF effect of IAT, examine the effect of covariates (socio-demographic and Internet use variables) on IAT latent factors.

As shown in Table 7, the model fit indices of CFA showed acceptable to good fit (RSMEA = 0.065, CFI = 0.954, TLI = 0.948), the factor loadings ranged from 0.487 to 0.814. The latent factors were significantly correlated to each other, ranged from 0.845 to 0.902.

**Table 7:** Factor loadings, factor correlation and fit indices of CFA model, MIMIC model, and MIMIC with DIF model by overall sample (*n* = 1131)

items	CFA model	MIMIC model	MIMIC with DIF model
Factor 1			
IAT1	0.683	0.677	0.687
IAT2	0.765	0.788	0.832
IAT5	0.682	0.673	0.683
IAT6	0.771	0.775	0.782
IAT8	0.814	0.827	0.871
Factor 2			
IAT10	0.692	0.686	0.685
IAT11	0.735	0.729	0.728
IAT12	0.661	0.662	0.631
IAT13	0.712	0.733	0.724
IAT14	0.601	0.592	0.590
IAT15	0.754	0.759	0.758
IAT16	0.742	0.755	0.754
IAT17	0.743	0.758	0.757
IAT18	0.700	0.723	0.722
IAT19	0.673	0.679	0.669
IAT20	0.765	0.775	0.774
Factor 3			
IAT3	0.718	0.720	0.720
IAT4	0.487	0.518	0.516
IAT7	0.687	0.694	0.695
IAT9	0.627	0.624	0.625
Factor correlation			
Factor2 WITH			
Factor1	0.845	0.815	0.815
Factor3 WITH			
Factor1	0.853	0.828	0.828
Factor2	0.902	0.889	0.889
**Model fit**			
RMSEA	0.065	0.042	0.040
90% C.I.	(0.061 0.069)	(0.039 0.045)	(0.037 0.042)
CFI	0.954	0.960	0.965
TLI	0.948	0.954	0.959

The result of MIMIC model showed that the 3-factor model of IAT with covariates fitted the data well (RMSEA = 0.040, CFI = 0.963, TLI = 0.957). The significant effect of Internet use covariates on the three latent factors were time spent online per day (Table 8), which was positively related to Factor 1 (*B* = 0.078, *p* = 0.000, *β* = 0.315), Factor 2 (*B* = 0.080, *p* = 0.000, *β* = 0.317), Factor 3 (*B* = 0.064, *p* = 0.000, *β* = 0.245).

**Table 8:** The impact of covariates on IAT latent factors and items

	MIMIC model				MIMIC model with DIF			
predictors	*B*	*S.E.*	*p*	*β*	*B*	*S.E.*	*p*	*Β*
**Factor 1**								
Female	−0.082	0.049	0.098	−0.117	− 0.081	0.049	0.099	−0.112
Age	−0.005	0.019	0.804	−0.016	−0.005	0.019	0.805	−0.016
Time	0.078	0.008	**0.000**	0.315**	0.099	0.008	**0.000**	0.388**
experience	0.006	0.007	0.381	0.039	0.006	0.007	0.381	0.037
Year 1	0.205	0.085	**0.016**	0.292*	0.204	0.084	**0.016**	0.283*
Year 2	0.217	0.072	**0.003**	0.309**	0.216	0.072	**0.003**	0.300**
Major in science	−0.049	0.067	0.469	− 0.070	− 0.049	0.067	0.467	− 0.068
Major in engineering	0.048	0.068	0.484	0.068	0.048	0.068	0.484	0.067
Major in others	−0.002	0.060	0.979	−0.003	−0.002	0.060	0.978	−0.003
General	−0.146	0.078	0.062	−0.208	−0.146	0.078	0.062	−0.202
Cellphone game	0.076	0.077	0.319	0.108	0.076	0.076	0.319	0.105
SNS	−0.008	0.062	0.900	−0.011	−0.008	0.062	0.900	−0.011
Other	0.107	0.080	0.182	0.153	0.107	0.080	0.182	0.148
**Factor2**								
female	−0.094	0.047	**0.047**	−0.132*	−0.065	0.047	0.171	−0.092
Age	−0.004	0.013	0.744	−0.015	−0.004	0.013	0.744	−0.015
time	0.080	0.008	**0.000**	**0.317**	0.077	0.008	**0.000**	0.305**
experience	0.004	0.005	0.420	0.027	0.004	0.005	0.419	0.027
Year 1	0.213	0.078	**0.006**	0.299**	0.213	0.078	**0.006**	0.300**
Year 2	0.261	0.070	**0.000**	0.367**	0.261	0.070	**0.000**	0.368**
Major in science	−0.022	0.066	0.739	−0.031	−0.022	0.066	0.738	−0.031
Major in engineering	−0.020	0.067	0.762	−0.028	−0.020	0.067	0.763	−0.028
Major in others	−0.032	0.058	0.573	−0.045	−0.033	0.058	0.573	−0.047
General	−0.175	0.073	**0.016**	−0.246*	−0.175	0.073	**0.016**	−0.247*
Cellphone game	0.132	0.074	0.076	0.186	0.132	0.074	0.076	0.186
SNS	−0.001	0.059	0.984	−0.001	0.001	0.059	0.985	0.001
Other	0.141	0.079	0.075	0.198	0.141	0.079	0.075	0.199
**Factor3**								
female	−0.111	0.059	0.061	−0.150	− 0.111	0.059	0.061	−0.150
Age	−0.010	0.016	0.510	−0.033	−0.010	0.016	0.510	−0.033
time	0.064	0.009	**0.000**	0.245**	0.064	0.009	**0.000**	0.245**
experience	0.000	0.007	0.962	−0.001	0.000	0.007	0.962	−0.001
Year 1	0.307	0.097	**0.002**	0.414**	0.306	0.097	**0.002**	0.412**
Year 2	0.250	0.085	**0.003**	0.337**	0.250	0.085	**0.003**	0.337**
Major in science	−0.054	0.083	0.516	− 0.073	−0.054	0.083	0.516	−0.073
Major in engineering	−0.046	0.082	0.575	−0.062	−0.046	0.082	0.575	−0.062
Major in others	0.036	0.072	0.618	0.049	0.036	0.072	0.619	0.048
General	−0.204	0.093	**0.028**	−0.275*	−0.204	0.093	**0.028**	−0.275*
Cellphone game	0.005	0.091	0.953	0.007	0.005	0.091	0.954	0.007
SNS	−0.065	0.075	0.385	−0.088	−0.065	0.075	0.385	−0.088
Other	0.035	0.095	0.713	0.047	0.140	0.097	0.148	0.189
**Testing DIF**								
female →IAT13					−0.358	0.067	0.000	−0.340**
time spent online →IAT2					−0.058	0.010	0.000	−0.160**
time spent online →IAT8					−0.054	0.011	0.000	−0.146**
time spent online → IAT12					0.039	0.011	0.000	0.103**
SNS → IAT12					0.333	0.077	0.000	0.309**
SNS → IAT19					−0.370	0.085	0.000	− 0.353**
Other → IAT4					−0.444	0.112	0.000	−0.434 **

*B* unstandardized estimate, *S.E.* standard error, *β *standardized estimate. *p < 0.05, **p < 0.01

The significant group difference on the latent factor scores were found on gender, Internet use group, and grade. Female users had 0.132 *SD* lower latent scores than male on Factor 2. Year 3 students had lower latent scores than year 1(0.292 SD, 0.299SD, and 0.414 SD for factor 1, 2 and 3 respectively) and year 2 students (0.309SD, 0.367SD, and0.337SD for factor 1, 2 and 3 respectively) on the all three latent factors. General users had 0.246 SD and 0.275 SD lower latent scores than MMORPG users on factor 2 and 3 respectively.

Differential item functioning (DIF) was tested by checking the modification indices (MI) which is the indication of significant association in the model from covariant to IAT items. As shown in Table 8, the final MIMIC model with DIF identified seven items displayed DIF and demonstrated good fit to data (RSMEA = 0.040, CFI = 0.965, TLI = 0.959). People spent more time online were more likely to endorse lower scores on two items that were IAT2(B = -0.054, *p* = 0.000, β = − 0.160) and IAT8 (B = -0.054, *p* = 0.000, β = − 0.146), while report higher scores on IAT12 (*B* = 0.039, *p* = 0.000, *β* = 0.103); female had decreased probability to endorse IAT13 (*B* = -0.358, *p* = 0.000, *β* = − 0.340) than male; SNS users were likely to endorse higher scores on IAT12 (*B* = 0.333, *p* = 0.000, *β* = − 0.309) and endorse lower scores on IAT19 (*B* = -0.370, *p* = 0.000, *β* = − 0.353); Other users prefer to endorse lower scores on IAT4 (*B* = -0.444, *p* = 0.000, *β* = − 0.434).

Comparing MIMIC model with DIF and without DIF on the regression coefficients of covariates to the latent factor (see Table 8), the significant change was on the effect of female to factor 2 (*β* was changed from-0.132 to − 0.092, with significant to non-significant). The other changes of regression coefficient were very small which did not contaminate the result of the association between covariates and three latent factors, such as the regression coefficient was increased slightly from time spent online to factor 1 (*β* was changed from 0.315 to 0.388), decreased from year 1 to factor 1 (*β* was changed from 0.292 to 0.283) (see Table 8).

## Discussion

The objective of the 1st phase study is to examine the item quality and factor structure of IAT (Chinses version). The original IAT is a 6-point rating scale. A study on a Greek version IAT suggested that 3-point rating scale performed better [25]. Another study in Malaysia suggested to keep the 6-point rating scale for a bilingual version IAT (English and Malay [41]. Rasch model analysis of this study first found the disordered threshold of 6-category rating scale which suggested to collapse 1 (rarely) and 2 (occasionally) response. The 5-point rating scale worked better and applied in 2nd phase study. The unidimensional structure of IAT was confirmed in this study that was consistent with the previous researches [25, 41]. There was no item with severe misfit that implied the item was productive for the measure. Overall, a good to excellent person and item separation (2.66 and 6.86) revealed that the Chinese version of IAT with 5-point rating scale is a reliable instrument to measure PIU.

A 3-factor solution of IAT was first identified in the 1st phase study sample and then confirmed by the 2nd phase study sample. The result of 3-factor structure was quite similar with study among Hong Kong university students [31] and Hong Kong adolescents [26, 27]. The possible reason is that those studies were held in different area of China; the research samples use same language and share similar culture. The major difference was on two items (IAT 7 and 11) which were dropped in study of Hong Kong [27, 31] as its poor performance in EFA (e.g. low factor loading), and kept in this study with its good item fit and high factor loadings. The improvement of item 7 may related to rephrase “email” to “online instant message (e.g. qq, wechat) in this study, as the word “email” may link to work which were found by researcher’s previous study in Malaysia [34]. Consistent with most studies, IAT 11 was not found any problem in this research. The difference may related to that Chang and Law (2008) set a higher cut-off point of factor loading (> 0.4), (31), while other researchers usually set a lower criteria (> 0.3) at the preliminary stage or EFA analysis so that the relevant item could be included, such as study on Greek adolescents [42], Italian adults [43], Thai university students [24]. A number of other influences may also affect the variance, such as translation, sample,culture,and data analysis method.

The MIMIC model in the 2nd phase study found significant DIF relating to 6 IAT items (IAT2, IAT4, IAT8, IAT12, IAT13, IAT19). Examing the effect of DIF on IAT latent factor found that only one itme (IAT13 snap, yell, or act annoyed if someone bothers you while you are online) loading on factor 2 (excessive use and emotional conflict of Internet use) made measurement bias on gender. The significant gender difference was no longer existed when correcting DIF effect, which implied that DIF was the main reason for gender difference on the factor 2 “excessive use and emotional conflict of Internet use”. This result was inconsistent with the study in Malaysian [34] which found IAT 14 performed DIF on gender, but did not contaminate any latent factor scores of IAT. It seems that male tended to more sensitive on IAT13 when they experienced with emotion symptoms of Internet use. Female in China may perform less observed emotion symptoms related to Internet use. Comparing MIMIC model with and without DIF indicated that the magnitude of DIF for the other 5 items was very limited and the effect on the latent factor scores of IAT was negligible. Item delete is not suggested as the effect size is limited to one latent factor scores of IAT, not on the other two and the item is important to measure emotional symptoms of internet overuse. DIF may be related to translation or culture. In addition, this is the first study to validate Chinese version of IAT in item level, the relevant academic evidence is very few under Chinese background. Modification on IAT13 relating to translation or expression may be necessary to control the measurement bias on gender.

In this study, the significant effect of covariates (socio-demographic and Internet use variables) on the 3 latent factors of IAT were time spent online, year 1, year 2, general users. Time spent online was significant predictor of all three IAT latent factors. It implied that students spent more time online could experience higher level of PIU symptoms. This result was consistent with most previous research findings that there were close relationship between duration of Internet use and PIU [34, 44–47]. This study found that college students spent 5.66 h (SD = 2.82) online per day. Comparing to the past researches in China found that time on daily Internet use is increasing among college and university students [48]. The popular of smartphone may play a role on the increasing time of Internet use as smartphone make it easy to access Internet. Students with PIU tended to spent more time online compared with non-PIU [49]. The impact of Internet first use in early age is inconsistent. Some studies found that the Internet use experience and the age of first Internet use was related to the level of PIU [34, 50], while other studies did not find the relation [44]. The result of this study did not found any significant relation between the Internet use experience and the three IAT latent factor scores.

Online games were deemed as more attractive than offline games [51, 52]. Tone, Zhao and Yan (2014) found the attraction of online games was the most important factor of PIU compared to other factors (personality, life events). And the MMORPG users were more likely to develop PIU than other game users [53, 54]. This study divided the Internet users into five groups (general, MMORPG, cellphone game, SNS, others) according to their self-report on the favorite Internet activities. The general users reported significant lower scores than MMORPG users on factor 2 and 3 of IAT, while the scores of the other three groups (cellphone game, SNS, others) did not find any significant difference with MMORPG users on the three IAT latent factors. It implied that the other Internet activities such as SNS users, cellphone game users, had the same risk of PIU as MMORPG users.

This study found that students in year 3 reported significantly lower scores than students in year 1 and year 2 on the all three latent factors of IAT. The result was different with the studies in Jiang Su [55] and Xin Jiang [56] China, which found that the students in year 2 and 3 were more vulnerable to PIU as they had less study work and more free time to get online. The inconsistent finding on grade may related to the sample which in this study were 3-year college student, while others were 4 or 5-year undergraduate students. The final year students were not included in the study of Jiang Su and Xin Jiang which only took the students in year1, 2 and 3 as their research sample. Li, Wang, & Wang, (2009) included the fourth year students and did not find any grade difference related to PIU [57]. The third year students in this study were in the final year of their college study. They were usually concentrated on their graduate project, internship and job searching, which may decrease the risk of PIU.

## Conclusion and future study

A 5-point scale is more adapted to the Chinese version of IAT. Item improvement was efficient that the problematic items found in literature was performed good in this study. The overall psychometric property of this Chinese version IAT was good with limited DIF effect in one item. One item need adaption to control the gender bias in the future study. Bigger sample size and equivalent sample across grade was suggested.

## Data Availability

The datasets generated and analyzed during the current study are not publicly available due to funding policy but are available from the corresponding author on reasonable request.

## References

[1] China Internet Network Information Center (2021). The 47th statistical report on the development of internet in China [internet].

[2] Shao YY, Xu S, Chen J (2020). Causes and outcomes of adolescent inter- net addiction and intervention effects. Chinese J Sch Heal.

[3] Trojak B, Zullino D, Achab S (2017). Brain stimulation to treat internet addiction: a commentary. Addict Behav.

[4] Iyitoglu O, Çeliköz N (2017). Exploring the impact of internet addiction on academic achievement. Eur J Educ Stud.

[5] Koo HJ, Kwon JH (2014). Risk and protective factors of internet addiction: a meta-analysis of empirical studies in Korea. Yonsei Med J.

[6] Akhter N (2013). Relationship between internet addiction and academic performance among university undergraduates. Educ Res Rev.

[7] Cui Y, Yang YT, Qian H, Cui W, Cui LJ (2020). Analysis on the related factors of college students’ network use and internet addiction. Med Res Educ.

[8] Yan W, Li Y, Sui N (2014). The relationship between recent stressful life events, personality traits, perceived family functioning and internet addiction among college students. Stress Health.

[9] Caplan SE (2002). Problematic internet use and psychosocial well-being: development of a theory-based cognitive–behavioral measurement instrument. Comput Hum Behav.

[10] Davis RA (2001). A cognitive-behavioral model of pathological internet use. Comput Hum Behav.

[11] Beard KW, Wolf EM (2001). Modification in the proposed diagnostic criteria for internet addiction. CyberPsychol Behav.

[12] Cash H, D. C, H. Steel A, Winkler A (2012). Internet addiction: a brief summary of research and practice. Curr Psychiatr Rev.

[13] Chou C, Hsiao M (2000). Internet addiction, usage, gratication, and pleasure experience: the Taiwan college students ’ case. Comput Educ.

[14] Young KS, Nabuco de Abreu C, Young KS, de Abreu CN (2011). Internet addiction: a handbook and guide to evaluation and treatment.

[15] Li L, Xu DD, Chai JX, Wang D, Li L, Zhang L (2018). Prevalence of internet addiction disorder in Chinese university students: a comprehensive meta-analysis of observational studies. J Behav Addict.

[16] Adiele I, Olatokun W (2014). Prevalence and determinants of internet addiction among adolescents. Comput Hum Behav.

[17] Dalbudak E, Evren C, Aldemir S, Evren B. The severity of internet addiction risk and its relationship with severity of borderline personality features, childhood traumas, dissociative experiences, depression and anxiety symptoms among Turkish University students. Psychiatry Res. 2014; [cited 2014 Jun 5]; Available from: http://www.sciencedirect.com/science/article/pii/S016517811400170X. 10.1016/j.psychres.2014.02.032 25023365

[18] González E, Orgaz B (2014). Problematic online experiences among Spanish college students: associations with internet use characteristics and clinical symptoms. Comput Hum Behav.

[19] Kuss DJ, Griffiths MD, Binder JF (2013). Internet addiction in students: prevalence and risk factors. Comput Hum Behav.

[20] Ng CG, Isa SM, Hashim AH, Pilla SK, Singh MKH. Validity of the Malay version of the internet addiction test: a study on a Group of Medical Students in Malaysia. Asia Pac J Public Health. 2012; [cited 2014 Apr 12]; Available from: http://www.ncbi.nlm.nih.gov/pubmed/22652253. 10.1177/1010539512447808 22652253

[21] Frangos CC, Frangos CC, Kiohos AP (2010). Internet addiction among Greek University Students : demographic associations with the phenomenon , using the Greek version of Young ’ s internet addiction test. Int J Econ Sci Appl Res.

[22] Laconi S, Rodgers RF, Chabrol H (2014). The measurement of internet addiction: a critical review of existing scales and their psychometric properties. Comput Hum Behav.

[23] Moreno MA, Jelenchick L, Cox E, Young H, Christakis DA (2011). Problematic internet use among US youth: a systematic review. Arch Pediatr Adolesc Med.

[24] Neelapaijit A, Pinyopornpanish M, Simcharoen S, Kuntawong P, Wongpakaran N, Wongpakaran T (2018). Psychometric properties of a Thai version internet addiction test. BMC Res Notes.

[25] Panayides P, Walker MJ (2012). Evaluation of the psychometric properties of the internet addiction test (IAT) in a sample of Cypriot high school students: the Rasch measurement perspective. Eur J Psychol.

[26] Lai CM, Mak KK, Cheng C, Watanabe H, Nomachi S, Bahar N (2015). Measurement invariance of the internet addiction test among Hong Kong, Japanese, and Malaysian adolescents. Cyberpsychol Behav Soc Netw.

[27] Lai C, Hil MP, Mak K, Watanabe H, Ang RP, Pang JS (2013). Psychometric properties of the internet addiction test in Chinese adolescents. J Pediatr Psychol.

[28] Jelenchick L, a, Becker T, Moreno M a. (2012). Assessing the psychometric properties of the internet addiction test (IAT) in US college students. Psychiatry Res.

[29] Korkeila J, Kaarlas S, Jääskeläinen M, Vahlberg T, Taiminen T (2010). Attached to the web--harmful use of the internet and its correlates. Eur Psychiatry.

[30] Widyanto L, Griffiths MD, Brunsden V (2011). A psychometric comparison of the internet addiction test, the internet-related problem scale, and self-diagnosis. Cyberpsychol Behav Soc Netw.

[31] Chang MK, Law SPM (2008). Factor structure for Young’s internet addiction test: a confirmatory study. Comput Hum Behav.

[32] Khazaal Y, Billieux J, Thorens G, Khan R, Louati Y, Scarlatti E (2008). French validation of the internet addiction test. CyberPsychol Behav.

[33] Ferraro G, Caci B, D’Amico A, Di Blasi M (2007). Internet addiction disorder: an Italian study. CyberPsychol Behav.

[34] Lu X, Yeo KJ, Guo F, Zhao Z (2020). Factor structure and a multiple indicators multiple cause model of internet addiction test: the effect of socio-demographic and internet use variables. Curr Psychol.

[35] Bond TG, Fox CM. Applying the Rasch Model : fundamental measurement in the human sciences second edition. New York: Lawrence Erlbaum Associates; 2007.

[36] Linacre PJM (2006). A User’s guide to winsteps ministep.

[37] Linacre JM (2011). Winsteps help for Rasch analysis.

[38] Harrington D. Confirmatory factor analysis (Google eBook): Oxford University Press; 2008. p. 136. [cited 2014 May 7]. Available from: http://books.google.com/books?id=PPbgH8fzwAUC&pgis=1

[39] Schermelleh-engel K, Moosbrugger H, Müller H (2003). Evaluating the fit of structural equation Models : tests of significance and descriptive goodness-of-fit measures. Methods Psychol Res Online.

[40] Hu LT, Bentler PM (1999). Cutoff criteria for fit indexes in covariance structure analysis: conventional criteria versus new alternatives. Struct Equ Model.

[41] Lu X, Yeo KJ (2015). Psychometric properties of the internet addiction test in a sample of Malaysian undergraduate students. Psicol Educ.

[42] Tsermentseli S, Karipidis N, Samaras P, Thompson T (2018). Assessing the factorial structure of the internet addiction test in a sample of Greek adolescents. Hell J Psychol.

[43] Faraci P, Craparo G, Messina R, Severino S. Internet addiction test (IAT): which is the best factorial solution? J Med Internet Res. 2013;15(10):e225. 10.2196/jmir.2935 PMC3806548 24184961

[44] Siste K, Suwartono C, Nasrun MW, Bardosono S, Sekartini R, Pandelaki J (2021). Validation study of the Indonesian internet addiction test among adolescents. PLoS One.

[45] Alejandro Tafur-Mendoza A, César Acosta-Prado J, Arturo Zárate-Torres R, Emilio R-OD. Assessing the psychometric properties of the internet addiction test in Peruvian University students. Int J Environ Res Public Heal Artic. 2020; [cited 2021 Aug 19]; Available from: www.mdpi.com/journal/ijerph. 10.3390/ijerph17165782 PMC7459878 32785063

[46] Durkee T, Kaess M, Carli V, Sarchiapone M, Wasserman C, Hoven C (2013). 1672 – pathological internet use among european adolescents: psychopathology and self-destructive behaviors. Eur Psychiatry.

[47] Gencer SL, Koc M (2012). Internet abuse among teenagers and its relations to internet usage patterns and demographics. Educ Technol Soc.

[48] Lu X (2019). Investigation of internet use among college students:motivation and internet apps. Chinese J ICT Educ.

[49] Hou Q, Zhang Z, Yang G (2013). Effects of different addiction states on activities, personalities and self-control Abilitiy among undergraduate internet users. J Zhejiang Univ (Science Ed).

[50] Black DW, Shaw M, Coryell W, Crowe R, McCormick B, Allen J (2015). Age at onset of DSM-IV pathological gambling in a non-treatment sample early- versus later-onset. Compr Psychiatry.

[51] Tone H-J, Zhao H-R, Yan W-S (2014). The attraction of online games: an important factor for internet addiction. Comput Human Behav.

[52] Kuss DJ, Griffiths MD (2011). Internet gaming Addiction : a systematic review of empirical research. Int J Ment Heal Addict.

[53] Lee M-S, Ko Y-H, Song H-S, Kwon K-H, Lee H-S, Nam M (2007). Characteristics of internet use in relation to game genre in Korean adolescents. Cyber Psychol Behav.

[54] Chappell D, Eatough V, N.O. Davies M, Griffiths M. EverQuest —It’s just a computer game right? An interpretative phenomenological analysis of online gaming addiction. Int J Ment Heal Addict 2006;4(3):205–216. [cited 2016 May 17]. Available from: http://download.springer.com/static/pdf/682/art%25253A10.1007%25252Fs11469-006-9028-6.pdf?originUrl=http%253A%252F%252Flink.springer.com%252Farticle%252F10.1007%252Fs11469-006-9028-6&token2=exp=1463465385~acl=%252Fstatic%252Fpdf%252F682%252Fart%2525253A10.1007%2525252Fs11469-006-902

[55] Wei L, Xiyan B, Bin W, Quan C (2010). Investigation on the internet dependence of undergraduates and analysis of correlative causes. Chinese Gen Pract.

[56] Ting G (2013). Study on the excessive internet use of a comprehensive UniversityCollege students.

[57] Li H, Wang J, Wang L (2009). A survey on the generalized problematic internet use in Chinese college students and its relations to stressful life events and coping style. Int J Ment Health Addict.

